# Semianalytical Solution for the Deformation of an Elastic Layer under an Axisymmetrically Distributed Power-Form Load: Application to Fluid-Jet-Induced Indentation of Biological Soft Tissues

**DOI:** 10.1155/2017/9842037

**Published:** 2017-03-08

**Authors:** Minhua Lu, Shuai Huang, Xianglong Yang, Lei Yang, Rui Mao

**Affiliations:** ^1^Guangdong Key Laboratory for Biomedical Measurements and Ultrasound Imaging, School of Biomedical Engineering, Shenzhen University, Shenzhen, China; ^2^College of Civil Engineering, Shenzhen University, Shenzhen, China; ^3^Guangdong Province Key Laboratory of Popular High Performance Computers, College of Computer Science and Software Engineering, Shenzhen University, Shenzhen, China

## Abstract

Fluid-jet-based indentation is used as a noncontact excitation technique by systems measuring the mechanical properties of soft tissues. However, the application of these devices has been hindered by the lack of theoretical solutions. This study developed a mathematical model for testing the indentation induced by a fluid jet and determined a semianalytical solution. The soft tissue was modeled as an elastic layer bonded to a rigid base. The pressure of the fluid jet impinging on the soft tissue was assumed to have a power-form function. The semianalytical solution was verified in detail using finite-element modeling, with excellent agreement being achieved. The effects of several parameters on the solution behaviors are reported, and a method for applying the solution to determine the mechanical properties of soft tissues is suggested.

## 1. Introduction

Indentation is one of the most commonly used methods to measure the mechanical properties (e.g., Young's modulus and Poisson's ratio) of biological soft tissues in situ or in vivo, such as articular cartilage [1, 2], the liver, human skin [3], and residual limbs [4], as it does not require special preparation for the specimens. Rigid cylindrical flat-ended or spherical indenters are often employed in both conventional and ultrasound indentation-based measurement techniques [5–9]. Once the loading force has been measured with a force sensor and the tissue deformation together with tissue thickness is recorded with an ultrasound transducer (in ultrasound indentation), Young's modulus and Poisson's ratio of a soft-tissue sample can be calculated from the relationship reported by Hayes et al. and other investigators [10–12]. However, the direct contact associated with the use of a stiff mechanical indenter may cause tissue damage, especially when the tissue has been in the degenerative conditions, and/or those organs, such as cornea, are not suitable for direct touch. Therefore, the use of noncontact devices is desirable whenever possible. 

Systems based on fluid-jet-induced indentation have been developed by many researchers for measuring the mechanical properties of soft tissues. The key idea of such systems is to use a fluid jet as an indenter that exerts a mechanical load on the soft tissues so as to avoid the shortcomings associated with direct contact between a rigid measurement instrument and soft tissues. Water and air are commonly used as the fluid mediums. Duda et al. [13] developed a device based on water-jet-induced indentation with optical modality to quantify the cartilage stiffness and demonstrated a strong correlation with standard indentation measurements. However, it cannot provide the tissue thickness which is a critical parameter to calculate Young's modulus of soft tissue from the indentation load-deformation curve. Lu et al. [14, 15] developed a noncontact indentation system utilizing the compression induced by a water jet and high-frequency ultrasound to measure the properties of soft tissues. They utilized high-frequency ultrasound to measure the initial thickness and dynamic deformation of tissues under water-jet loading. Their tests on phantoms showed that the system was able to quantify the elastic properties of soft tissues and had the capability for elasticity mapping of the tissues in a C-scan test. The system has also been employed to assess degeneration of articular cartilage [1]. Huang and Zheng [16] further designed a miniaturized water-jet- and ultrasound-based indentation system. Their results showed that the indentation system produced results comparable to those obtained using the conventional one and that it was able to characterize the integrity of articular cartilage under arthroscopic control.

Prompted by the inconvenience of water spillage when using a water jet to induce indentation inside the body, Huang et al. [17] developed an air-jet-based indentation system based on optical coherence tomography for measuring the mechanical properties of soft tissues. They performed experiments both on silicone phantoms and on the human hand in vivo, with the results demonstrating the capacity of the system to detect biomechanical changes in soft tissues. The air-jet-based indentation system was subsequently used by Chao et al. to characterize the biomechanical properties of the forefoot plantar soft tissue of different ages [18] and to measure the stiffness of the healing wounds in rat skin [19]. However, they found that the stiffness measured by the air-jet system differed greatly from those mechanical properties measured by the tensile testing machine.

In most of the above-reviewed researches, a stiffness coefficient (SC) was defined to interpret the loading-deformation data of the soft tissues:

(1) $$\begin{array}{c} SC=\frac{F}{d}\cdot \frac{L_{0}}{A}, \end{array}$$

where *F* is the total force applied on the specimen by the fluid jet, *A* is the area of the outlet of the fluid nozzle, and *L*_0_ and *d* are the initial thickness and deformation of the specimen, respectively. The definition looks very similar to the compressive Young's modulus of soft tissue measured by unconfined axial compression. However, it should be noted that the total force in (1) was calculated from the fluid pressure in the pipe supplying the fluid because the distribution of the impacting pressure of the fluid jet upon the specimen is complicated and difficult to measure directly. Moreover, the deformation of the specimen induced by a fluid jet also varies spatially across the sample surface, and the sensitivity depends on the lateral resolution of the ultrasound beam. The values of *d* and *A* are both ambiguous to some extent, and hence the calculated stiffness coefficient is essentially only a nominal value. The relationship among SC, Young's modulus, and Poisson's ratio is also not obvious. In general, obtaining Young's modulus and Poisson's ratio from the indentation induced by an impinging fluid jet would require a calibration to be performed based on the conventional contact indentation. This requirement would be highly inconvenient when a fluid jet is used to induce indentation in vivo. Therefore, quantifying the mechanism of soft-material deformation due to compression caused by an impinging fluid jet will benefit the application of fluid-jet-induced indentation to clinical diagnosis.

The classical problem of an elastic half-space indented by a rigid indenter was investigated using theoretical analysis and finite-element simulation. Exact solutions in the form of force-indentation relationships, contact pressure distributions, and stress and displacement fields exist for axisymmetric indenter geometries (e.g., cylinder, sphere, and cone) [12, 20, 21]. The effects of many other parameters, including friction between the indenter and the soft tissue, model geometry, substrate deformability, and curvature, on the calculation of Young's modulus from indentation response of soft tissue were also studied [10, 22–25].

From a mathematical point of view, the indentation induced by contact with a conventional stiff object (e.g., cylinder or sphere indenter) differs significantly from that induced by a fluid jet. In the former, the deformation where the specimen touches the cylindrical flat-ended or spherical punch can be easily measured and it is always treated as a known condition in a theoretical analysis. However, in the latter, even the deformation of the specimen can be measured by an ultrasound transducer; the distribution of the impacting load applied by the fluid jet is unknown. In fact, the wall pressure (i.e., the fluid pressure on the surface of specimen) induced by the fluid jet is greatly affected by the scale (i.e., Reynolds number) as well as the boundary conditions (e.g., the nozzle geometry and the distance from the nozzle outlet to the target surface) [26]. Generally, the wall pressure follows a Gaussian profile for a two-dimensional jet but not for a three-dimensional jet [27]. Boyer et al. [3] applied classical contact mechanics to model the wall pressure by a function with a power form and achieved excellent agreement between the theoretically calculated pressure and the experimentally measured pressure on a rigid plate. Their solutions were used to assess the effects of ageing on the mechanical properties of skin in vivo with their air-jet-based indentation system. However, the solutions they used are derived from elastic materials with infinite thickness, and hence they might not be valid in most clinical applications where the thickness of the soft tissues is finite (and sometimes very small). This means that it is important to determine the analytical solution for the deformation of elastic materials with finite thickness compressed by an impinging fluid jet.

This study employed a semianalytical method to solve the equation describing the indentation induced by a fluid jet. The soft tissue was assumed to be a homogeneous, isotropic elastic layer with finite scale in thickness and infinite size in extent. It was overlaid on a rigid foundation. The wall pressure was modeled by a power-form function as reported by Boyer et al. [3]. To validate the solutions, finite-element modeling (FEM) was performed using a static structural module in the ANSYS software. The results of the analytical solutions and FEM were compared in detail.

## 2. Mathematical Analysis

For homogeneous, isotropic materials, when the body forces and inertial effects are neglected and the deformation is small, the equilibrium equations of the linear theory of elasticity can be expressed in terms of the displacement vector as

(2) $$\begin{array}{c} \left(\;1-2\nu \;\right)\nabla^{2}u+\nabla \left(\;\nabla \cdot u\;\right)=0, \end{array}$$

where **u** is the displacement vector, *ν* is Poisson's ratio of soft tissue, and ∇ is the gradient operator.

**Figure 1:**
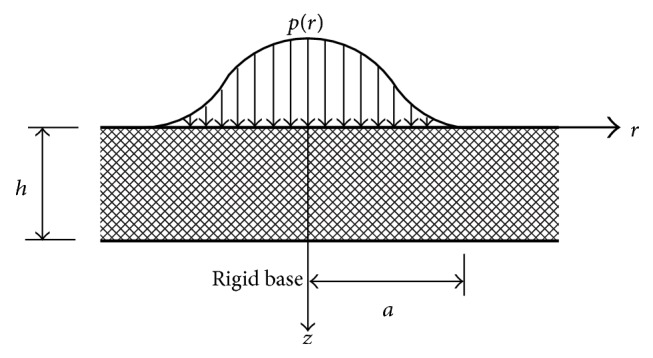
Schematic diagram of the deformation of an elastic layer under an axisymmetrically distributed load.

Considering the equilibrium of an infinite elastic layer with thickness *h* adhering to an immovable rigid base, the layer deformation under an axisymmetrically distributed load from the impingement of a circular fluid jet is axisymmetric and so can be analyzed in a cylindrical coordinate system. A schematic diagram of this problem is shown in Figure 1.

The considered problem can be solved conveniently by using the Boussinesq-Papkovich potential functions for the components of the displacement vector; that is,

(3) 2Gur=−∂∂rΦ0+zΦ1,2Guz=−∂Φ0∂z−z∂Φ1∂r+3−4νΦ1,

where *G* = *E*/2(1 + *ν*) is the elastic shear modulus, *E* is Young's modulus, (*r*, *θ*, *z*) are the radial, tangential, and axial coordinates in a cylindrical coordinate system, (*u*_*r*_, 0, *u*_*z*_) are the radial, tangential, and axial components of the displacement vector, respectively, and Φ_0_ and Φ_1_ are harmonic functions, which are written in the form

(4) Φ0=∫0∞Aλshλh−z+Bλchλh−z·J0λrdλ,Φ1=∫0∞Cλshλh−z+Dλchλh−z·J0λrdλ,

where* A*,* B*,* C*, and *D* are functions of *λ* to be determined, *J*_0_(*λr*) is the Bessel function of the first kind of order zero, and axial-direction coordinate *z* in the elastic layer ranges from 0 to* h*.

The normal and tangential stress components are given in terms of the potentials as

(5) σzz=21−ν∂Φ1∂z−∂2Φ0∂z2−z∂2Φ1∂z2,σrz=∂∂r1−2νΦ1−∂Φ0∂z−z∂Φ1∂z.

The elastic layer is indented on by a prescribed normally distributed load at the surface and the layer adheres to an immovable rigid base, and so the following boundary conditions apply:

(a) At the surface,* z* = 0:
  (6) σzz=pr,σrz=0,0≤r≤∞,
where *p*(*r*) is a prescribed load function of radial coordinate,* r*.
(b) At the bottom,* z* =* h*:
  (7) $$\begin{array}{c} u_{r}=u_{z}=0,\;\left(0\leq r\leq \infty \right). \end{array}$$

By using (3)–(5) and boundary conditions (6) and (7) and following the same procedures as described by Hayes et al. [12], the following integral equation can be obtained:

(8) $$\begin{array}{c} \overset{\infty }{\underset{0}{\int }}C^{\prime }\left(\;\lambda \;\right)J_{0}\left(\;\lambda r\;\right)d\lambda =p\left(\;r\;\right),\;\left(0\leq r\leq \infty \right). \end{array}$$

The axial component of the displacement at the layer surface (*z* = 0), which is a parameter of interest in many situations, can be given by

(9) G1−vuzr,0=∫0∞C′λMλJ0λrdλ,0≤r≤∞,

where

(10) $$\begin{array}{c} M\left(\;\lambda \;\right)=\frac{1}{\lambda }\left[\;\frac{\left(3-4\nu \right)sh\left(\lambda h\right)ch\left(\lambda h\right)-\lambda h}{{\left(\lambda h\right)}^{2}+4{\left(1-\nu \right)}^{2}+\left(3-4\nu \right)sh^{2}\left(\lambda h\right)}\;\right]. \end{array}$$

When *p*(*r*) is known, (8) may be used to determine *C*′(*λ*), and then displacement *u*_*z*_ at the layer surface (*z* = 0) can be obtained using (9).

In practical applications, it is convenient to introduce the dimensionless variables *α* = *λh* and *r*′ = *r*/*h*. After nondimensionalizing the load function by an appropriate constant *P* as *p*′(*r*′) = *p*(*r*)/*P*, (8) and (9) become

(11) ∫0∞QαJ0αr′dα=p′r′,0≤r′≤∞,

(12) G1−vuzr′,0hP=∫0∞1αQαM′αJ0αr′dα,0≤r′≤∞,

where

(13) Qα=C′λhP,M′α=3−4νshαchα−αα2+41−ν2+3−4νsh2α.

In the study of Boyer et al. [3], the pressure of an impinging circular fluid jet was modeled by a function of the power form

(14a) $$\begin{array}{c} p\left(\;r\;\right)=\left\{\begin{array}{cc} p_{0}a^{-2\left(m-1/2\right)}{\left(a^{2}-r^{2}\right)}^{m-1/2} & \left(0\leq r<a\right),\\ 0 & \left(r\geq a\right), \end{array}\right. \end{array}$$

where *a* is the radius of the area over which jet is impinging, *p*_0_ is the pressure at the center point of that area, and *m* is an integer. These three parameters depend on the nozzle geometry, the Reynolds number of the fluid jet, and the distance between the nozzle outlet and the tissue surface. There are two reasons why this form of pressure distribution was chosen: (i) this distribution fits the pressure of an impinging circular fluid jet with acceptable accuracy and (ii) according to Boussinesq theory there exist analytical solutions for the deformation of an elastic half-space under this form of load [28].

As mentioned above, function (14a) is convenient for deriving the analytical solution for the deformation of an elastic half-space (with infinite thickness), but it is not convenient for the deformation problem of an elastic layer with finite thickness. To derive the analytical solution for the deformation of an elastic layer, the pressure function is modified slightly as

(14b) $$\begin{array}{c} p\left(\;r\;\right)=\left\{\begin{array}{cc} p_{0}a^{-2\left(m-1\right)}{\left(a^{2}-r^{2}\right)}^{m-1} & \left(0\leq r<a\right),\\ 0 & \left(r\geq a\right). \end{array}\right. \end{array}$$

**Figure 2:**
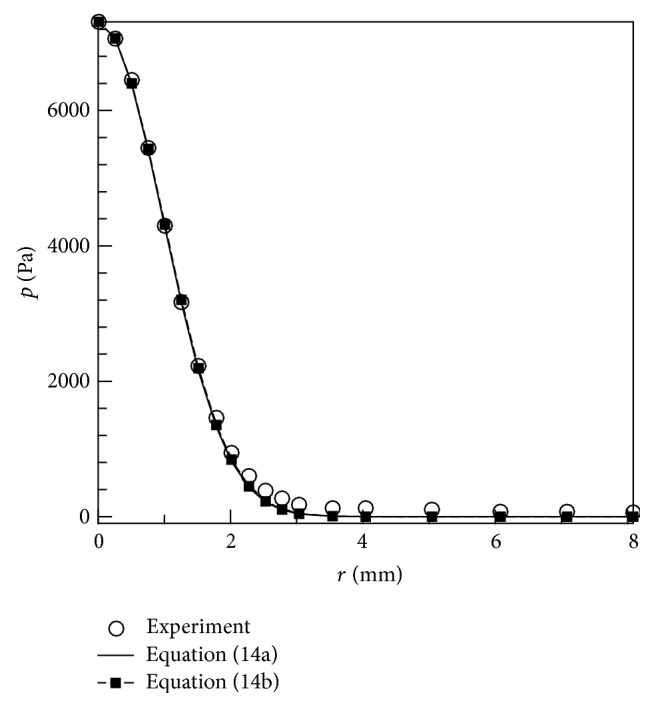
Comparison of the fitted curves and experimental data [3].

Integer *m* may in general be sufficiently large (e.g.,* m* ≥ 28) [3] for the difference between (14a) and (14b) to be small.  Figure 2 shows the fitted curves based on (14a) and (14b) and the experimental data that correspond to the case with a flow rate of 20 L_n_/min and* a* = 8 mm [3]. The figure illustrates that the difference is acceptably small.

Introducing the dimensionless variable *r*′ = *r*/*h*, (14b) becomes

(15) pr=p0ha2m−1ah2−r′2m−1=Pp′r′,0≤r′<ah,0r′≥ah,

where

(16) P=p0ha2m−1,p′r′=ah2−r′2m−1,0≤r′<ah.

Using the Heaviside unit step function [29], namely,

(17) $$\begin{array}{c} H\left(\;\frac{a}{h}-r^{\prime }\;\right)=\left\{\begin{array}{cc} 1 & \left(0\leq r^{\prime }<\frac{a}{h}\right)\\ 0 & \left(r^{\prime }\geq \frac{a}{h}\right), \end{array}\right. \end{array}$$

and inserting (16) into (11) yield

(18) ∫0∞QαJ0αr′dα=ah2−r′2m−1Hah−r′.

Letting *Q*′(*α*) = *Q*(*α*)/*α*, (18) becomes

(19) ∫0∞αQ′αJ0αr′dα=ah2−r′2m−1Hah−r′.

Using the Hankel integral transform [29], *Q*′(*α*) can be solved as

(20) $$\begin{array}{c} Q^{\prime }\left(\;\alpha \;\right)=2^{\left(m-1\right)}\Gamma \left(\;m\;\right){\left(\;\frac{a}{h}\;\right)}^{m}\alpha^{-m}J_{m}\left(\;\frac{a}{h}\alpha \;\right), \end{array}$$

and then

(21) $$\begin{array}{c} Q\left(\;\alpha \;\right)=\alpha Q^{\prime }\left(\;\alpha \;\right)=\alpha 2^{\left(m-1\right)}\Gamma \left(\;m\;\right){\left(\;\frac{a}{h}\;\right)}^{m}\alpha^{-m}J_{m}\left(\;\frac{a}{h}\alpha \;\right), \end{array}$$

where Γ(*m*) = (*m* − 1)! is the factorial function and *J*_*m*_((*a*/*h*)*α*) is the Bessel function of the first kind of order* m*.

Inserting (21) into (12) and rearranging it yield

(22) uzr′,0=p0h1−ν2EFv,m,ah,r′,0≤r′≤∞,

where

(23) Fv,m,ah,r′=ah2−m2mΓm·∫0∞1αmM′αJmahαJ0αr′dα.

This equation is a function of Poisson's ratio *ν*, fitted integer* m*, the ratio of the radius of the area over which the jet is impinging to the thickness of the elastic layer (*a*/*h*), and dimensionless radius *r*′. Equation (22) indicates that the axial displacement is proportional to the pressure at the center point of the fluid jet and inversely proportional to Young's modulus of the elastic layer, while it varies nonlinearly with other quantities. The infinite integral on the right-hand side of (23) includes the product of two Bessel functions of the first kind with different orders, and it can be integrated numerically using the adaptive Gaussian quadrature [30]. However, we found that if the Bessel functions are calculated sufficiently accurately, Gauss-Laguerre quadrature is also sufficiently accurate and efficient when *m* is not too small (e.g.,* m* > 3) because the integrand in the infinite integral goes rapidly to zero as the argument increases.

The Bessel function can be calculated using its integral representation

(24) $$\begin{array}{c} J_{m}\left(\;x\;\right)=\frac{1}{\pi }\overset{\pi }{\underset{0}{\int }}\cos\left(\;m\theta -x\sin\theta \;\right)d\theta . \end{array}$$

This integral can be calculated using Gauss-Legendre quadrature. However, the integral will be unstable when *m* is large (e.g.,* m* > 10) for small* x*, leading to an incorrect value of the infinite integral on the right-hand side of (23), which is also calculated using Gauss-Laguerre quadrature. Alternatively, the Bessel function can be calculated using its infinite-power-series expansion form

(25) Jmx1Γm+1x2m·1−Z11−Z21−Z31−⋯,

where

(26) $$\begin{array}{c} Z_{k}=\frac{1}{k\left(k+m\right)}{\left(\;\frac{x}{2}\;\right)}^{2},\;\left(k=1,2,3,\ldots \right). \end{array}$$

The Bessel function calculated using (25) is stable when *m* is large and *x* is small, but it may be incorrect for large *x* because in practice only finite terms can be considered, so that the truncation error becomes nonnegligible with large *x*. Additionally, it is also limited by the machine precision when *m* and *x* are both large due to the presence of* x*^*m*^ term.

We used a combination algorithm to calculate Bessel function *J*_*m*_(*x*); namely, (25) with 50 terms was used for *x* ≤ *m*/2, while (24) with Gauss-Legendre quadrature was used for *x* > *m*/2. It was found that this combination algorithm works well over wide ranges of the values of *m* and* x*, and it was sufficiently accurate for our purposes.

In applications involving using a fluid jet to apply indentation on an elastic layer, the maximum axial displacement, which generally occurs at the center point on the layer surface (*r*′ = 0; *z* = 0), is more useful and easier to obtain by various measurement techniques, such as ultrasound, optical coherence tomography, and other contact methods. Using the property of *J*_0_(0) = 1, it can be derived that

(27) $$\begin{array}{c} u_{zmax}=u_{z}\left(\;0,0\;\right)=\frac{p_{0}h\left(1-\nu^{2}\right)}{E}\kappa \left(\;v,m,\frac{a}{h}\;\right), \end{array}$$

where

(28) κv,m,ah=ah2−m2mΓm∫0∞1αmM′αJmahαdα

is a scaling factor that depends on Poisson's ratio of the elastic layer *ν*, fitted integer* m*, and aspect ratio* a*/*h*.

## 3. Finite-Element Modeling

**Figure 3:**
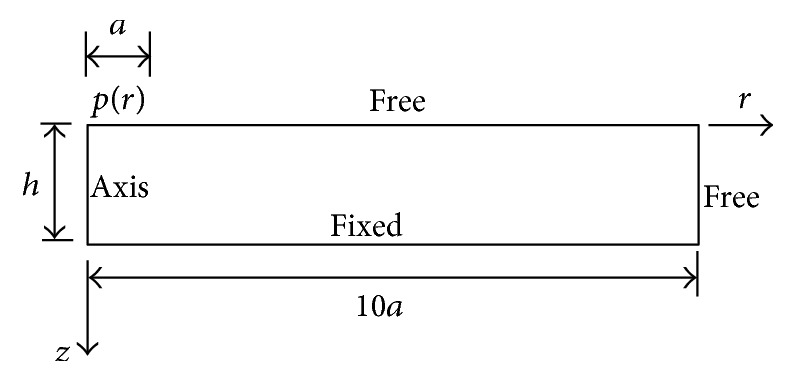
Geometry and boundary conditions for finite-element modeling (FEM).

To verify the validity of the analytical solutions obtained above, an axisymmetric finite-element model was established using a static structural module in ANSYS. The material was assumed to be linear, homogeneous, and isotropic (with constant Young's modulus and Poisson's ratio), and both the deformation and strain were assumed to be small. The model geometry and boundary conditions are shown in Figure 3. The computational domain is a rectangle with dimension 10*a* in the radial direction and *h* in the axial direction. The left side of the domain is an axis boundary, the bottom side has a fixed boundary, a normal pressure according to (14b) acts upon the top surface at 0 ≤* r* ≤* a*, and the other boundaries are free. Since the stresses are much larger near a contact point [3], distance 10*a* is sufficiently far from the lateral boundary condition to ensure that its effects can be neglected.

Block-meshing technology was applied to ensure the accuracy of the FEM and reduce the computational costs. Uniformly distributed meshes with a grid size of 0.01*a* were applied in the radial direction at 0 ≤* r* ≤* a*, and bias-type distributed meshes with a bias factor of 30 and a total number of 200 were used elsewhere. Uniform meshes with a grid size of 0.01*a* (the total number of meshes varied with the layer thickness,* h*) were used in the axial direction. It was verified that mesh-independent solutions were obtained when using this mesh density.

## 4. Results and Discussion

This section analyzes the solution of the model described above and discusses its applications in a fluid-jet-based indentation system. To facilitate our analyses, we decided to select one case as a base for further comparisons. The parameters for this case were as follows, which were comparable with those experimental values from the ultrasound water-jet indentation system [14]: thickness of the elastic layer (*h*), 0.004 m; radius of the area of the impinging fluid jet (*a*), 0.01 m; Young's modulus (*E*), 1.0 × 10^6^ Pa; and Poisson's ratio (*ν*), 0.3. The pressure at the center point (*p*_0_) was 2.0 × 10^4^ Pa, and fitted integer *m* was 30.

**Figure 4:**
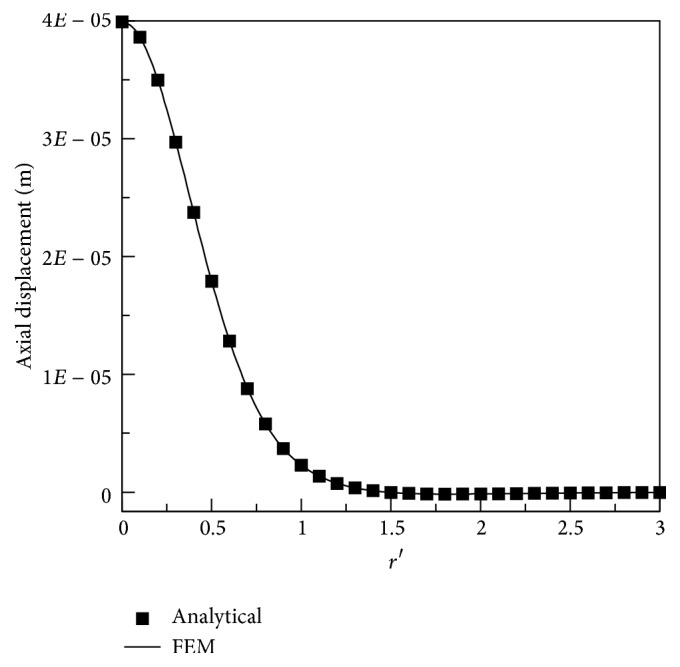
Axial displacements at the surface (*z* = 0) from the analytical solution and FEM, where* r*′ =* r*/*h*.

The axial displacements at the surface (*z* = 0) obtained from the analytical solution using (22) and FEM are compared in Figure 4. It is obvious that excellent agreement was achieved.

To further verify the validity of the analytical solutions, more cases were calculated with a wide range of parameters. For simplicity, we report here only the maximum axial displacement, *u*_*z*max_. Equation (27) indicates that the relationships among *u*_*z*max_, *p*_0_, and *E* are very simple; namely, *u*_*z*max_ is proportional to *p*_0_ and inversely proportional to *E*. However, the relationships among *u*_*z*max_,* a*,* h*,* m*, and*ν* are complex and need to be considered in more detail.  Table 1 lists the values of *u*_*z*max_ computed using (27) and simulated using FEM with constant* a* = 0.01 m and different* h*,* m*, and*ν* values. The table indicates that the analytical solutions agree very well with results from FEM over a wide range of parameters. Generally, the difference between analytical and simulated results increases with increasing fitted integer *m* and Poisson's ratio*ν* and decreasing *h*. The maximum difference was no more than 0.07%, which occurred for* h* = 0.001 m,* m* = 40, and*ν* = 0.5.

**Figure 5:**
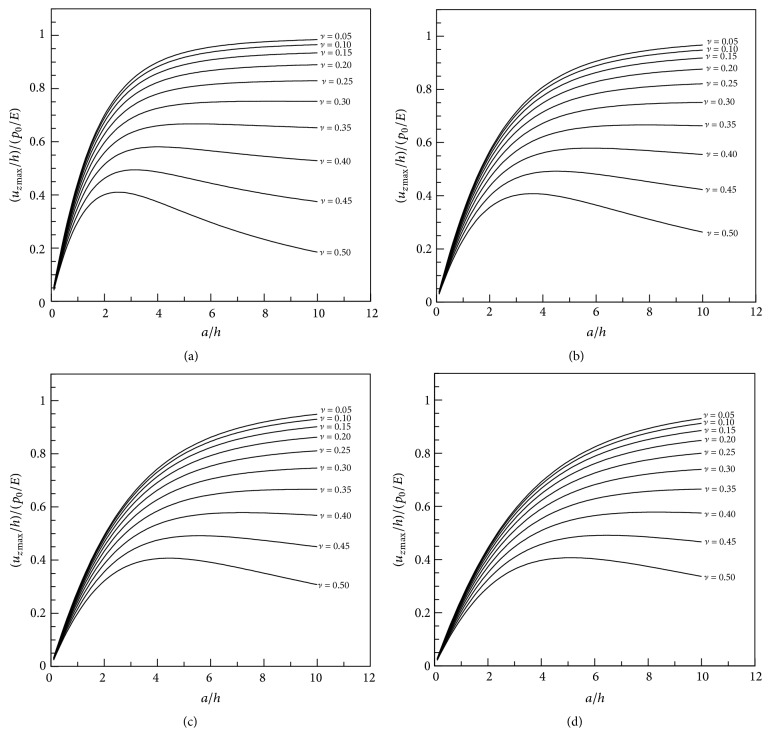
Nondimensional indentation versus aspect ratio for different Poisson's ratios with *m* values of (a) 10, (b) 20, (c) 30, and (d) 40.

To show how different parameter values affect the axial displacements, the nondimensional indentation, defined as the ratio of the nondimensional maximum axial displacement to nondimensional pressure (*u*_*z*max_/*h*)/(*p*_0_/*E*) [12], is plotted against* a*/*h* for different *m* and*ν*values in Figure 5. It is obvious from the figure that when the aspect ratio (*a*/*h*) is small, the indentation varies markedly with this ratio, whereas Poisson's ratio has no significant effect. In contrast, when the aspect ratio is large, Poisson's ratio has a marked effect, while the aspect ratio has only a slight effect. It can be predicted that the indentation for each Poisson's ratio will tend to a limit as the aspect ratio increases, because this is similar to the problem of a thin layer being compressed by a large indenter, which means that the edge effects will be negligible. The indentations have similar features irrespective of the value of *m*. For small Poisson's ratios, the indentation increases with* a*/*h*, while for large Poisson's ratios the indentation first increases and then decreases as* a*/*h* increases further.

In fluid-jet-based indentation applications, (27) can be used to calculate Young's modulus. Rearranging that equation yields

(29) $$\begin{array}{c} E=\frac{p_{0}h\left(1-\nu^{2}\right)}{u_{zmax}}\kappa \left(\;v,m,\frac{a}{h}\;\right), \end{array}$$

where parameters *p*_0_,* a*, and *m* only depend on characteristics of the fluid-jet instrument, namely, the nozzle geometry, the Reynolds number of the fluid jet, and the distance from the nozzle outlet to the target surface. These characteristics can be determined before the indentation tests are performed through mechanical measurements. Tissue thickness *h* can also be measured before indentation tests using ultrasound nondestructively or a needle punch which is a destructive method after indentation. Poisson's ratio of soft tissue is often considered to be constant in indentation tests or can be measured by ultrasound or mechanical methods [14]. The maximum axial displacement at the surface of the elastic layer, *u*_*z*max_, can be recorded during the tests by various approaches such as spatial sensors or ultrasonically. Knowledge of these parameters allows scaling factor*κ* to be calculated using (28), and then Young's modulus of the material can be calculated directly using (29).

Most soft tissues, such as articular cartilage, liver, and human skin, are viscoelastic materials, and so, in general, dynamic analysis should be used. However, as predicted by Hayes et al. [12], the theory based on elastic materials can be used in some limited cases, such as in creep tests, to predict the instantaneous elastic response under a step load. With the closed-form solution (e.g., the scaling factor) in the present analysis, a parametric analysis of the tests of fluid-jet-induced indentation can be easily carried out. Unfortunately, direct comparisons between the present theory and the experimental measurements reported in the literature [3, 13, 14, 17] are not possible, since the effects of applying a distributed pressure to the specimen surface using a fluid jet have not been measured. Future studies should investigate the behaviors of fluid jets and their effects on soft tissues.

## 5. Conclusions

To measure or image the mechanical properties of tissues has been attracting increasing research efforts during the recent decades. The stiffness of soft tissues may change under different pathologic situations, such as sclerosus cancer, edema, degeneration, fibrosis [31]. Normal tissues may also have different stiffness, which is important information for tissue characterization. The mechanical properties of tissues can have different values depending on whether they are measured in vivo or in vitro and in situ or as an excised specimen [32, 33]. During recent decades, ultrasound techniques together with compression, vibration, or indentation have been successfully used for the measurement or imaging of the mechanical properties of soft tissues, especially for the musculoskeletal tissues [34–39].

Our group previously developed a noncontact ultrasound indentation system for quantitative measurement of tissue stiffness [14]. The advantage of this technique is that it utilized water jet as a “soft indenter” instead of a rigid indenter to compress the soft tissue so as to avoid potential damage caused by a rigid indenter which may result in stress concentration at the edge of the indenter [10]. However, the loading during the water-jet indentation is not easy to measure directly; therefore the intrinsic Young's modulus of soft tissue cannot be derived during the water-jet ultrasound indentation, which hinders the application of this technique.

In this study, a mathematical model for testing fluid-jet-induced indentation has been developed, and its semianalytical solution was determined. After detailed verification with FEM was carried out, the effects of altering the values of several parameters on the solution behavior were further analyzed. The following conclusions can be drawn from the results obtained:

1. The axial displacement of the elastic layer, *u*_*z*_, is proportional to the pressure at the center point of the fluid jet, *p*_0_, and inversely proportional to Young's modulus of the elastic layer,* E*, while it varies nonlinearly with other quantities.
2. When* a*/*h* is small, Poisson's ratio has only a slight effect on the nondimensional indentation; however, when* a*/*h* is large, Poisson's ratio has a significant effect.
3. Parameters *p*_0_,* a*,* m*,* h*, and*ν*, can be determined before performing tests of fluid-jet-induced indentation. Combining with *u*_*z*max_ recorded during the indentation tests, Young's modulus of materials can be calculated directly by the analytical solution. It should be noted that the pressurized area* a* would need to be determined beforehand for various injection velocities and separation distance. In this study, it is assumed to be the same as the radius of the fluid-jet nozzle, ranging from 1.5 mm to 2 mm, based on our previous experimental experiences. We usually keep the distance between the fluid-jet nozzle and the tissue surface at around 5 mm, and the injection velocity changes from 0 to 15 m/s within 1 second for an indentation test on soft tissue. However, the pressurized area *a* would be largely different from the nozzle radius when the nozzle is far from the tissue surface, especially when the injection velocity is low.

It wound be of interest to comment on the tissue model used in our analysis. The soft tissue in this study was modeled as a thin layer of linear elastic, homogeneous, and isotropic material bonded to a rigid fixed substrate to simulate the boundary conditions of articular cartilage. However, the nonlinear and viscoelastic properties of soft tissues have not been addressed. Furthermore, instead of single-phase model to describe the soft tissue, the nonlinearity and viscoelasticity of soft tissues have been investigated using biphasic and triphasic models [40, 41]. In these models, the intrinsic fluid load support during indentation has been verified during the indentation by a rigid indenter. Whether the fluid-jet indentation would have significant effect on the soft tissue when the soft tissue is modeled as multiphasic material is a critical issue to be further analyzed.

## Figures and Tables

**Table 1:** Maximum axial displacement (*u*_*z*max_, ×10^−5^ m) values obtained from the analytical solution and FEM with different parameters.

*h* (m)						
*ν*	0.001		0.01		0.02	
	Analytical	FEM	Analytical	FEM	Analytical	FEM
*m* = 10						
0.1	1.9303	1.9301	8.8642	8.8638	10.019	10.019
0.2	1.7795	1.7794	8.5055	8.5052	9.6679	9.6677
0.3	1.5035	1.5034	7.9206	7.9202	9.0892	9.0890
0.4	1.0574	1.0573	7.0899	7.0895	8.2731	8.2730
0.5	0.3691	0.3690	5.9795	5.9791	7.2017	7.2016

*m* = 20						
0.1	1.8959	1.8955	6.6904	6.6899	7.2850	7.2847
0.2	1.7529	1.7525	6.4408	6.4403	7.0400	7.0398
0.3	1.5021	1.5017	6.0317	6.0313	6.6354	6.6351
0.4	1.1104	1.1102	5.4532	5.4527	6.0660	6.0657
0.5	0.5261	0.5259	4.6875	4.6871	5.3227	5.3225

*m* = 30						
0.1	1.8602	1.8597	5.6252	5.6247	6.0257	6.0255
0.2	1.7247	1.7242	5.4230	5.4425	5.8269	5.8267
0.3	1.4924	1.4920	5.0909	5.0904	5.4981	5.4978
0.4	1.1366	1.1362	4.6219	4.6214	5.0357	5.0354
0.5	0.6153	0.6149	4.0043	4.0038	4.4338	4.4334

*m* = 40						
0.1	1.8250	1.8244	4.9577	4.9572	5.2598	5.2595
0.2	1.6963	1.6957	4.7835	4.7830	5.0882	5.0879
0.3	1.4788	1.4783	4.4969	4.4963	4.8041	4.8038
0.4	1.1498	1.1493	4.0926	4.0920	4.4050	4.4046
0.5	0.6732	0.6727	3.5617	3.5611	3.8861	3.8857

## References

[1] Lu M.-H., Zheng Y. P., Huang Q.-H. (2009). Noncontact evaluation of articular cartilage degeneration using a novel ultrasound water jet indentation system. *Annals of Biomedical Engineering*.

[2] Chen X., Zimmerman B. K., Lu X. L. (2015). Determine the equilibrium mechanical properties of articular cartilage from the short-term indentation response. *Journal of Biomechanics*.

[3] Boyer G., Pailler Mattei C., Molimard J., Pericoi M., Laquieze S., Zahouani H. (2012). Non contact method for in vivo assessment of skin mechanical properties for assessing effect of ageing. *Medical Engineering and Physics*.

[4] Mak A. F. T., Zhang M., Tam E. W. C. (2010). Biomechanics of pressure ulcer in body tissues interacting with external forces during locomotion. *Annual Review of Biomedical Engineering*.

[5] Zheng Y. P., Choi Y. K. C., Wong K., Chan S., Mak A. F. T. (2000). Biomechanical assessment of plantar foot tissue in diabetic patients using an ultrasound indentation system. *Ultrasound in Medicine and Biology*.

[6] Mow V. C., Gibbs M. C., Lai W. M., Zhu W. B., Athanasiou K. A. (1989). Biphasic indentation of articular cartilage—II. A numerical algorithm and an experimental study. *Journal of Biomechanics*.

[7] Arokoski J. P. A., Hyttinen M. M., Helminen H. J., Jurvelin J. S. (1999). Biomechanical and structural characteristics of canine femoral and tibial cartilage. *Journal of Biomedical Materials Research*.

[8] Athanasiou K. A., Fleischli J. G., Bosma J. (1999). Effects of diabetes mellitus on the biomechanical properties of human ankle cartilage. *Clinical Orthopaedics and Related Research*.

[9] Kawchuk G. N., Fauvel O. R., Dmowski J. (2000). Ultrasonic quantification of osseous displacements resulting from skin surface indentation loading of bovine para-spinal tissue. *Clinical Biomechanics*.

[10] Zhang M., Zheng Y. P., Mak A. F. T. (1997). Estimating the effective Young's modulus of soft tissues from indentation tests—nonlinear finite element analysis of effects of friction and large deformation. *Medical Engineering and Physics*.

[11] Jurvelin J., Kiviranta I., Säämänen A.-M., Tammi M., Helminen H. J. (1990). Indentation stiffness of young canine knee articular cartilage—influence of strenuous joint loading. *Journal of Biomechanics*.

[12] Hayes W. C., Keer L. M., Herrmann G., Mockros L. F. (1972). A mathematical analysis for indentation tests of articular cartilage. *Journal of Biomechanics*.

[13] Duda G. N., Kleemann R. U., Bluecher U., Weiler A. (2004). A new device to detect early cartilage degeneration. *American Journal of Sports Medicine*.

[14] Lu M. H., Zheng Y. P., Huang Q. H. (2005). A novel noncontact ultrasound indentation system for measurement of tissue material properties using water jet compression. *Ultrasound in Medicine and Biology*.

[15] Lu M.-H., Zheng Y.-P., Huang Q.-H. (2007). A novel method to obtain modulus image of soft tissues using ultrasound water jet indentation: A Phantom Study. *IEEE Transactions on Biomedical Engineering*.

[16] Huang Y. P., Zheng Y. P. (2010). Development and phantom test of a minimized water-jet ultrasound indentation system for arthroscopic measurement of articular cartilage integrity. *6th World Congress of Biomechanics (WCB 2010). August 1–6, 2010 Singapore*.

[17] Huang Y.-P., Zheng Y.-P., Wang S.-Z., Chen Z.-P., Huang Q.-H., He Y.-H. (2009). An optical coherence tomography (OCT)-based air jet indentation system for measuring the mechanical properties of soft tissues. *Measurement Science and Technology*.

[18] Chao C. Y. L., Zheng Y.-P., Huang Y.-P., Cheing G. L. Y. (2010). Biomechanical properties of the forefoot plantar soft tissue as measured by an optical coherence tomography-based air-jet indentation system and tissue ultrasound palpation system. *Clinical Biomechanics*.

[19] Chao C. Y. L., Ng G. Y. F., Cheung K.-K., Zheng Y.-P., Wang L.-K., Cheing G. L. Y. (2013). In vivo and ex vivo approaches to studying the biomechanical properties of healing wounds in rat skin. *Journal of Biomechanical Engineering*.

[20] Sakamoto M., Li G., Hara T., Chao E. Y. S. (1996). A new method for theoretical analysis of static indentation test. *Journal of Biomechanics*.

[21] Costa K. D., Yin F. C. P. (1999). Analysis of indentation: implications for measuring mechanical properties with atomic force microscopy. *Journal of Biomechanical Engineering*.

[22] Lu M. H., Zheng Y. P. (2004). Indentation test of soft tissues with curved substrates: a finite element study. *Medical and Biological Engineering and Computing*.

[23] Galbraith P. C., Bryant J. T. (1989). Effects of grid dimensions on finite element models of an articular surface. *Journal of Biomechanics*.

[24] Argatov I. I., Sabina F. J. (2016). Small-scale indentation of an elastic coated half-space: the effect of compliant substrate. *International Journal of Engineering Science*.

[25] Lin D. C., Dimitriadis E. K., Horkay F. (2007). Robust strategies for automated AFM force curve analysis—I. Non-adhesive indentation of soft, inhomogeneous materials. *Journal of Biomechanical Engineering*.

[26] Xu Z., Hangan H. (2008). Scale, boundary and inlet condition effects on impinging jets. *Journal of Wind Engineering and Industrial Aerodynamics*.

[27] Guo Y., Wood D. H. (2002). Measurements in the vicinity of a stagnation point. *Experimental Thermal and Fluid Science*.

[28] Johnson K. L., Johnson K. L. (2001). *Contact Mechanics*.

[29] Debnath L., Bhatta D. (2007). *Integral Transforms and Their Applications*.

[30] Lucas S. K. (1995). Evaluating infinite integrals involving products of Bessel functions of arbitrary order. *Journal of Computational and Applied Mathematics*.

[31] Greenleaf J. F., Fatemi M., Insana M. (2003). Selected methods for imaging elastic properties of biological tissues. *Annual Review of Biomedical Engineering*.

[32] Fung Y. C., Skalak R. (1981). Biomechanics: mechanical properties of living tissues. *Journal of Biomechanical Engineering*.

[33] Mow V. C., Hayes W. C. (1997). *Basic Orthopaedic Biomechanics*.

[34] Fatemi M., Greenleaf J. F. (1998). Ultrasound-stimulated vibro-acoustic spectrography. *Science*.

[35] Deffieux T., Gennisson J.-L., Tanter M., Fink M. (2008). Assessment of the mechanical properties of the musculoskeletal system using 2-D and 3-D very high frame rate ultrasound. *IEEE Transactions on Ultrasonics, Ferroelectrics, and Frequency Control*.

[36] Zheng Y.-P., Mak A. F. T. (1996). An ultrasound indentation system for biomechanical properties assessment of soft tissues in-vivo. *IEEE Transactions on Biomedical Engineering*.

[37] Ophir J., Céspedes I., Ponnekanti H., Yazdi Y., Li X. (1991). Elastography: a quantitative method for imaging the elasticity of biological tissues. *Ultrasonic Imaging*.

[38] Gennisson J. L., Cornu C., Catheline S., Fink M., Portero P. (2005). Human muscle hardness assessment during incremental isometric contraction using transient elastography. *Journal of Biomechanics*.

[39] Mao R., Zhang P., Li X., Liu X., Lu M. (2016). Pivot selection for metric-space indexing. *International Journal of Machine Learning and Cybernetics*.

[40] Mak A. F., Lai W. M., Mow V. C. (1987). Biphasic indentation of articular cartilage-I. Theoretical analysis. *Journal of Biomechanics*.

[41] Wang Q., Zheng Y. P., Niu H. J., Mak A. F. T. (2007). Extraction of mechanical properties of articular cartilage from osmotic swelling behavior monitored using high frequency ultrasound. *Journal of Biomechanical Engineering*.

